# Understanding torquetenovirus (TTV) as an immune marker

**DOI:** 10.3389/fmed.2023.1168400

**Published:** 2023-06-13

**Authors:** Edmund J. Gore, Lilli Gard, Hubert G. M. Niesters, Coretta C. Van Leer Buter

**Affiliations:** Department of Medical Microbiology, University Medical Center Groningen, University of Groningen, Groningen, Netherlands

**Keywords:** torquetenovirus, transplantation, TTV, immune marker, functional immunity, pitfalls

## Abstract

Torquetenovirus (TTV), a small, single stranded anellovirus, is currently being explored as a marker of immunocompetence in patients with immunological impairment and inflammatory disorders. TTV has an extremely high prevalence and is regarded as a part of the human virome, the replication of which is controlled by a functioning immune system. The viral load of TTV in plasma of individuals is thought to reflect the degree of immunosuppression. Measuring and quantifying this viral load is especially promising in organ transplantation, as many studies have shown a strong correlation between high TTV loads and increased risk of infection on one side, and low TTV loads and an increased risk of rejection on the other side. As clinical studies are underway, investigating if TTV viral load measurement is superior for gauging antirejection therapy compared to medication-levels, some aspects nevertheless have to be considered. In contrast with medication levels, TTV loads have to be interpreted bearing in mind that viruses have properties including transmission, tropism, genotypes and mutations. This narrative review describes the potential pitfalls of TTV measurement in the follow-up of solid organ transplant recipients and addresses the questions which remain to be answered.

## Introduction

Torquetenovirus (TTV) is currently being explored as a functional marker of the immune system in patients with immunological impairment and inflammatory disorders. The discovery of TTV in 1997, and subsequently other members of the anelloviridae family, have led to the concept of “commensal viruses”(1), defined as constituents of the human virome, not known to cause pathology in humans. TTV has an extremely high prevalence and has been detected in various conditions involving immune dysfunction and immune activation, including congenital and iatrogenic immunodeficiency, chronic viral infections and aging (2–6). The replication of TTV, similar to all the viruses constituting the virome, is controlled by a functioning immune system. And quantifying the virus load in blood is thought to be a potential read-out of this functionality. Nearly all studies investigating TTV in infectious and inflammatory processes show a correlation between unfavorable outcome or disease progression, and increasing or higher TTV loads (Tables 1, 2).

**TABLE 1:** Examples of infectious diseases in which TTV has been investigated as a prognostic marker.

Infectious disease	Outcome
BK viremia or BK virus nephropathy	TTV correlates with BK viremia but does not predict disease outcome in liver transplant recipients Herrmann et al. (57) In the first year after transplantation TTV viral load does not correspond to BK viral load and cannot be used as a predictive tool for the development of BK nephropathy Handala et al. (58) There is a weak correlation between BK viremia and TTV Fernández-Ruiz et al. (59)
EBV	The relationship between TTV and EBV viremia is controversial with some studies reporting a correlation, Garbuglia et al. (60), Borkosky et al. (61) and Mallet et al. (62) while others have reported no relationship between TTV and EBV viremia [Nordén et al. (41)]
CMV	TTV loads shortly after hematopoietic stem cell transplantation predict CMV DNAemia Albert et al. (63)
HIV	Baseline TTV plasma concentrations and CD4+ cell count are predictive of immune recovery after starting HAART Schmidt et al. (64)
Hepatitis C	TTV viral load is higher in patients with only a hepatitis infection compared to HIV/HCV coinfection. There is, however, no relationship between TTV viremia and the chance of HCV treatment response to DAA Lapa et al. (65)

**TABLE 2:** Examples of inflammatory diseases in which TTV has been investigated as a prognostic marker.

Disorder	Outcome
Asthma	Elevated TTV is seen in the nasal secretions of children with asthma compared to children without asthma Pifferi et al. (66)
Rheumatoid arthritis	TTV is lower in RA Maggi et al. (67). There is no difference in between TTV viral loads in patients with or without RA Seemayer et al. (68). TTV lower in patients receiving biological therapy than patients receiving conventional DMARD therapy Martín-López et al. (69). TTV levels 3 months after starting anti-TNF therapy are predictive of a disease reduction at month 6 Studenic et al. (45)

A biomarker of the immune status is especially promising in solid organ transplantation, where it could improve the lives of recipients (7). Antirejection therapy, which is essential for maintaining a transplanted organ, has side-effects and increase the recipient’s risk of developing an infection (8). Currently, antirejection medication is adjusted in response to trough levels of these drugs in blood. However, trough levels do not reliably predict rejection or infection in patients (9). A biomarker of the immune status would provide greater insight into the biological effects of the immunosuppressive drugs, and reduce the dangers of suboptimal dosing. Several potential biomarkers have been investigated in recent years, including T-cell subsets, T-cell proliferation assays and soluble CD30 (10–12). TTV is the most investigated biomarker in this context, with most studies showing that high TTV loads are associated with an increased risk of infection, and low TTV loads are associated with an increased risk of rejection (Table 3) (3). TTV viral load measurement in blood has advantages as a PCR can be done in a few hours, is affordable, and easy to perform. Moreover, stored blood samples can be tested, meaning that TTV measurements may also be used to obtain data retrospectively (13).

**TABLE 3:** Examples of studies which have shown that TTV is an immune marker in solid organ transplantation.

Transplanted organ	Studies that associate low TTV with rejection	Studies that associate high TTV with infection
Kidney	Schiemann et al. (70) Solis et al. (42) Fernández-Ruiz et al. (43) Strassl et al. (71) Doberer et al. (72) van Rijn et al. (39) Doberer et al. (73)	Maggi et al. (44) Strassl et al. (74) Solis et al. (42) Fernández-Ruiz et al. (43) Fernández-Ruiz et al. (59) Doberer et al. (72)
Lung	Görzer et al. (75) Jaksch et al. (40) Frye et al. (76)	Görzer et al. (77) Jaksch et al. (40) Frye et al. (76)
Liver	Simonetta et al. (78) Ruiz et al. (79)	Maggi et al. (44) Herrmann et al. (57) Ruiz et al. (79)

Two important clinical trials are currently being conducted, examining whether TTV viral load is superior to trough level measurements of the calcineurin inhibitor tacrolimus, when adjusting the dose of antirejection medication. The VIGILung Trial (Clinical trials number NCT04198506) is a multicenter study initiated in Germany and Austria in lung transplantation patients. This study was set up to investigate if a reduction in nephrotoxicity in the first year after transplantation can be achieved. 144 patients are randomized to undergo tacrolimus dose adjustments either based on TTV levels, or based on trough levels. The VIGILung trial is due to end in 2025 (14).

The second clinical trial is the TTV-GUIDE IT study, which is a multicenter European study in sixteen transplantation centers. This study was designed to explore if TTV can be safely used to adjust the dose of tacrolimus in kidney transplantation patients (EU CT-Number 2022-500024-30-00). A total of 260 patients are be randomized in to two groups. One group will have their tacrolimus dose adjusted based on TTV viral load, while the other group will have the tacrolimus adjusted based on tacrolimus trough levels. Patients will be followed for 13 months after transplantation with infection, allograft rejection, graft-loss and death as the primary outcomes. The TTV GUIDE IT study is due to end in August 2025 (15).

However, before TTV quantification can be implemented as a more reliable alternative to trough level measurements, there are some aspects that have to be considered. After all, in contrast with medication levels, when TTV loads are interpreted, virological features including transmission, tropism, mutations, viral variations and epidemiology, should be taken into account, as these may be relevant in the follow-up of solid organ transplant recipients (SOTR).

## Virology of TTV

**FIGURE 1:**
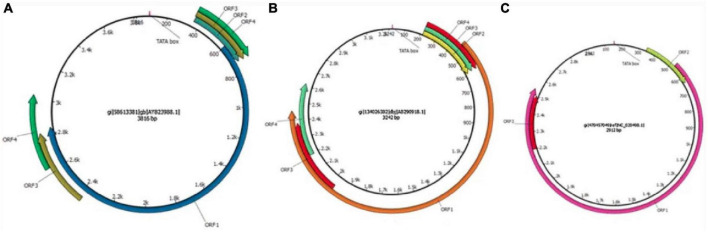
Schematic illustration of the genome organization of torque teno viruses. **(A)** TTV isolate 2 h (GenBank accession no. AY823988), **(B)** TTMDV isolate MD1-073 (GenBank accession no. AB290918), **(C)** TTMV isolate TTMV_LY1 (GenBank accession no. NC_020498.1). The arrows represent open reading frames. Used with permission (16).

Torquetenovirus is a small, non-enveloped, circular, negative-sense single stranded DNA anellovirus. The virus features an enormous sequence diversity, with so far five identified genogroups and over 50 genotypes. Sequence divergence within the family is high, as these five genogroups display at least 50% nucleotide divergence, and the different genotypes up to 30% divergence. The coding region of the TTV genome contains at least four open reading frames (ORFs), of which one contains three hypervariable regions. The untranslated region (UTR) is well preserved between the genotypes and represents a target for detection PCRs. TTV was the first identified member of the anellovirus family, however, more recently, torque teno minivirus (TTMV) and torque teno midivirus (TTMDV) have been identified in 2000 and 2007, respectively. In addition, several anelloviruses have been described in other mammals (Figure 1) (16, 17).

Torquetenovirus is thought to induce long term or chronic infections, with no known negative effects. The virus is found in many clinical syndromes where inflammation is part of the process, but also in the general population TTV can be detected in 2–90% of individuals (17). Studies in infants and children have found that transmission occurs early in life, likely occurring via breastmilk, saliva, the fecal-oral route, or through respiratory and droplet transmission. In addition, transmission in a healthcare setting is conceivable, as blood transfusions are a recognized transmission route. Longitudinal studies in infants showed that nearly all were infected with TTV before the age of 4 (18–21). Moreover, coinfections with different genotypes are common. Two different genotypes can be detected in most individuals and some individuals are co-infected with multiple genotypes, belonging to up to four different genogroups (18, 21).

## Replication of TTV

As with most viruses with a circular genomes it is believed that TTV replicates using rolling circle replication and escapes from infected cells through cell lysis (22, 23) It is as yet unclear precisely which cells are responsible for TTV replication. It was first reported from *in vitro* experiments in 2002, that activated PBMCs produce TTV (24). Longitudinal studies in hematology and transplantation patients showed subsequently that T- lymphocytes are the main source of TTV in peripheral blood (25–28). It was observed that cellular depletion resulting from conditioning for stem cell transplantation (SCT), resulted in a temporary loss of nearly all TTV in peripheral blood (25, 26). As lymphocytes start to reappear at first engraftment, TTV increases rapidly until reaching a peak at 60 days after SCT, after which a gradual decline is observed paralleling immune-reconstitution. In SOTR, many of whom have detectable TTV prior to transplantation, TTV loads decrease significantly in the first few days after induction immunosuppression therapy with T-lymphocyte inhibitors. Focosi et al. reported that T-cell depletion with Anti Thymocyte Globulin (ATG) in kidney transplant recipients induced a stronger decrease in TTV load than the less potent T-cell inhibitor basiliximab (27). Also, a higher ATG dose had a greater effect on the TTV load than a lower dose. Görzer et al. described a similar early decrease of the TTV load in lung transplant recipients (LTR) after induction with alemtuzumab (28). In this very early post-transplantation period, low or negative TTV loads should clearly not be regarded as a reflection of a functional immune system, but rather an effect of profound T-cell lymphopenia. After the initial period, TTV loads increased rapidly in all these cases, reflecting an immunosuppressive status.

## TTV DNA prevalence in materials and populations

Besides blood, TTV can be detected in many tissues and fluids, to such an extent that it is thought to be pantropic. Some researchers have reported fairly high viral loads in breastmilk, respiratory secretions, and saliva (29, 30). Some caution has to be taken in interpreting these sometimes conflicting results as many of these studies were carried out with different PCR assays resulting in higher or lower detection limit. There is no official standard yet to calibrate quantitative tests, and until recently, there was no commercial assay (31). Some research teams developed their own tests to detect and quantify the virus, with different sensitivities (29). In light of the enormous genotypic diversity of TTV and the various sensitivities of the PCR tests used to detect the virus, quantitative results are difficult to compare, not only between materials, but also populations. As such, healthy blood donors were found to have detectable TTV loads varying between 4% of cases in Iran to 96% of cases in Jordan. Within Europe, where many TTV-related studies are conducted, prevalence among healthy donors ranged between 46% in Italy and 76% in neighboring Austria, when tested with laboratory developed PCR tests (6, 32–34). A large virome study using whole genome sequencing, which is known to be less sensitive, showed that 14% of individuals from the USA had detectable TTV (35). There is not only a difference in the prevalence of TTV worldwide but the prevalence of certain genotypes also appears to change depending on the population studied (13, 16). The development of a commercial PCR test will make comparisons between studies, and thereby between populations and even different patient materials, much easier in future. Newly developed external quality control schemes, developed by QCMD (Quality Control in Molecular Diagnostics, Glasgow, Scotland, UK) as well as controls used in studies, should enable more standardization in viral load measurements within clinical studies.

## The immune response and TTV

Investigations into both the replication cycle of TTV as well as the immune response inhibiting replication, have undoubtedly been hampered by the fact that culturing this virus has been extremely difficult with few groups reporting success (22, 36). The almost ubiquitous nature of the virus also does not allow for comparisons between individuals who are carriers of active or dormant TTV replication in blood. Therefore the consequences of the antigenic pressures of this virus in infected individuals, or the absence of antigenic pressure in uninfected individuals has not been explored. Rocchi et al. showed that unmethylated CpG DNA of TTV is capable of inducing an inflammatory response through activation of TLR-9 *in vitro* (37). No studies have confirmed this finding *in vivo*, but longitudinal studies in which the acquisition of primary TTV infections and acquisition of new genotypes have been followed, do not seem to point toward a systemic inflammatory response to these infections (18, 21). As more evidence is accumulating that rejection and some infections after SOT are correlated with the TTV load measured in blood, the thought that the immunological pathways involved in these processes may overlap with the immune response against TTV is appealing. More research into these immunological pathways is urgently needed. In addition, greater understanding of how the various immunosuppressants impact the TTV load, is essential for any study investigating the use of the virus in the follow-up of SOTR.

In lung and kidney transplantation, the standard antirejection regimen is a combination of three drugs, consisting of a calcineurin inhibitor [mostly tacrolimus (TAC) or cyclosporine], a proliferation inhibitor [usually mycophenolate mofetil (MMF) or azathioprine (AZA), and prednisolone] (38). Alternative regimens may include mammalian target of rapamycin inhibitors (mTOR inhibitors) and belatacept. Immediately after transplantation, induction of immunosuppression may incorporate T-lymphocyte inhibitors such as ATG, alemtuzumab and basiliximab. Despite the above recommendations following SOT there is much variation in the regimes chosen by transplantation centers due to the availability of medication and inter-center culture. Further changes in the regimen during the follow-up period are usually motivated by side effects or other adverse events experienced by the patient. These practices explain the absence of large studies investigating TTV loads in patients groups of similar time after transplantation, in which different antirejection regimens are given in equal proportions to different study cohorts. Comparisons between different agents and their separate effects on the TTV load, have to be made by comparing either different transplantation centers with different standard regimens, or by comparing patient cohorts spanning a longer period of time, during which the standard regimen changed. This is exemplified by Görzer et al. who investigated the TTV load during the immediate post-transplantation period, in which all of the 46 lung transplantation recipients received alemtuzumab conditioning (28). Focosi et al. compared induction with basiliximab and ATG in kidney and pancreas transplantation, but in this study no one received alemtuzumab (27). van Rijn et al. reported the TTV load in a series of kidney transplantation patients in which the regime changed from cyclosporin-based to tacrolimus-based, during the study period (39). Jaksch et al. followed 143 LTRs, of which most patients (*n* = 133) received alemtuzumab conditioning (40). And because many of these studies were conducted with different TTV detection PCRs, the differential effect of the antirejection drugs are difficult to assess, even more so in the long term. To date, some studies have shown that TTV loads are relatively higher in tacrolimus-based regimens, as opposed to those based on cyclosporine, whereas others could not confirm this (41–44). Belatacept is not frequently used in any of the studies, but transplant patients receiving this agent seem to have lower TTV loads than those with TAC-based regimens (45). The same could be true for patients receiving mTOR inhibitors (46). Future studies aiming to use TTV to gauge the antirejection therapy should answer the question how much which agent has to be increased or decrease for the TTV load to move toward a desired level.

## The significance of a negative TTV PCR

There are multiple ways for a SOTR to become infected with TTV. The patient comes into contact with multiple sources of TTV, through the donation of the organ and through blood products if they are given. Finding a SOTR with no detectable TTV in blood therefore should be an extremely rare event, considering the immunosuppression induced by the antirejection therapy. Some cross-sectional and longitudinal studies nevertheless show that considerable numbers of SOTR may have a negative PCR at various timepoints during the follow up period (39, 47). The interpretation of a single negative sample or a series of samples from a particular patient is difficult, as there are several possible explanations. It could be the case that most of these patients have suppression of TTV replication due to a highly functional immune system. But there are several reasons, other than immunological control, why TTV may be undetectable. Extreme lymphopenia, the most obvious cause for TTV-negativity, can easily be excluded. When a reasonable number of circulating lymphocytes is present in the blood, several other explanations remain for a not detecting TTV. Firstly: the test in use fails to detect the genotypes infecting this individual, this is a theoretical possibility. With five genogroups known, and 29 genotypes of TTV found, it is conceivable that assays are not able to detect all genotypes. The commercial assay from Biomerieux is able to detect eight genotypes (31). Secondly, a PCR test may be negative simply because a patient has failed to become infected with TTV. Studies such as done by Görzer et al. show that all 143 LTR included in the study develop positive TTV loads within weeks after transplantation (28). Yet, even the most prevalent viruses are unable to infect the entire population. If a study were conducted to investigate the use of TTV after SOT, these rare individuals, along with the ones that are negative because their genotypes are not detected, could easily be identified and excluded from this type of follow-up. Knowing that nearly all SOTR will become TTV positive after induction of antirejection treatment, after the initial lymphopenic phase, TTV could be used as a marker of immunosuppression used in the majority of SOTR.

The third reason for a negative TTV test not implying immunological control, is the possibility that the immune system could clear a TTV infection. If it is possible that an individual clears TTV, than a TTV load would also remain negative, even after increasing the immunosuppression. The viral load would no longer be suitable as a marker for immunosuppression. One case reports details the appearance and disappearance of a TTV genogroup 2 infection, which could be an example of an infection which was subsequently cleared (48). Since there is no way to distinguish these virus-clearing patients from elite controllers, this would represent a conceivable risk.

## Significance of a high TTV load

Just as being TTV DNA -negative may not always mean someone is an elite TTV controller, there are reasons why a high TTV load may not always reflect a bad immune response. The main uncertainty is that it is not at all clear how new infections with different genotypes are dealt with by the immune system. A longitudinal study in children showed that the acquisition of new genotypes resulted in recurrent high viral loads (18). Moreover, Maggi et al. showed that individuals carrying more TTV genogroups had higher viral loads in general (20). These studies also suggest that it is a relatively common occurrence to become infected with new genotypes. The study in children shows that five out of five children acquired new genotypes during the study period. Maggi et al. showed that three out of three stem cell transplantation recipients were infected with new genogroups after SCT (21).

## The question of the ideal TTV load

Despite the potential for both highly positive and negative TTV loads not necessarily representing immunological control, multiple studies have shown a strong link between high TTV load and the risk of infection, as well as a relationship between low TTV load and the risk of rejection (Table 3). These studies were recently summarized by van Rijn et al., who conducted a meta-analysis (3). The definition of what constitutes a high and low TTV load varies depending on the method used, type of organ transplantation and period of observation. Nearly all the research on this subject was carried out by a limited number of research groups which, for a long time, used their own test, with their own definitions of a high or al low viral load. The correlation between low TTV levels and rejection seems to be stronger than the correlation between high TTV levels and infection risk (3). A major drawback in the studies is that the follow-up period is restricted to, in most cases, one or two years after transplantation. When observing the data, it is important to reflect on the type of infections that are usually seen during this very early time frame after transplantation and whether these infections are the result of the immunosuppression (8). Studies investigating TTV viral load versus infection risk, frequently investigate infections which are prominent in particular SOT populations, but that are only in part due to the immunosuppression. CMV infections are bound to occur when an organ from a seropositive donor is transplanted into a seronegative recipient. CMV reactivations in SOTR who were seropositive prior to transplantation may be caused by the degree of immunosuppression, but not all authors have concluded this (3, 39, 44). Urinary tract infections are particularly common after kidney transplantation because of urological problems resulting from new urinary tract connections made during the operation. Also, BK virus, which is found to correlate with immunosuppression in kidney transplant recipients (KTR) (49), is very uncommon in thoracic organ recipients, in spite of a higher dosing of immunosuppressants and median TTV load found in the latter group (50, 51). Infections which occur in all types of SOTR are more likely the result of immunosuppression, as is the case in chronic norovirus infections (52). These infections are nevertheless too infrequent to be statistically relevant, and they may occur at any time after transplantation, when recipients are no longer followed by the transplantation center.

**FIGURE 2:**
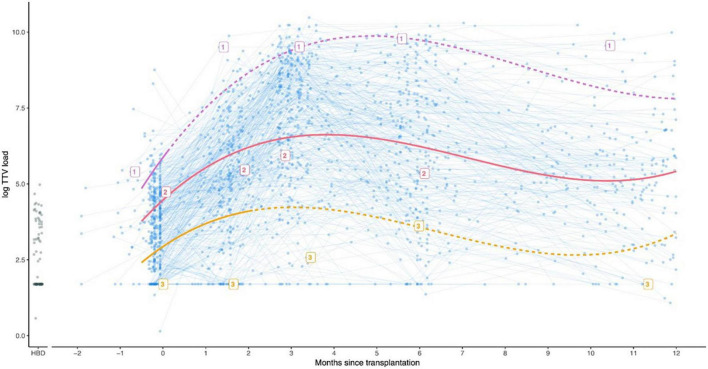
Graph showing the observed TTV loads for kidney transplant recipients (blue), and a group of 88 healthy blood donors for comparison (HBD, shown in black circles). In addition, three example patients indicated with the labels 1, 2, and 3 are shown. Patient 1 (purple) developed rejection 5 days after transplantation and was treated accordingly with a course of strong immunosuppressants. Patient 2 (pink) did not develop rejection. Patient 3 (orange) developed rejection 64 days after transplantation. Used with permission (39).

A rapidly increasing TTV load after transplantation leading to a peak in the first months, is universally seen in studies. Furthermore, observations during the first years after transplantation imply that the TTV peak is followed by a plateau and thereafter a slow reduction in TTV (Figure 2) (47). In contrast, cross-sectional studies suggest that SOTR who are further away from the transplantation date have lower TTV loads than those who were transplanted more recently. This was shown in research by our team in KTR with a follow-up period of up to 30 years after transplantation. The TTV load was significantly lower in KTR longer after transplantation. The study also showed that infections and the mortality rate due to an infectious cause were higher in patients with high TTV load, whereas we were not able to show a correlation between low TTV load and the risk for rejection (47). Likewise, a study in lung transplant recipients (LTR) showed that the TTV load was lower in recipients who were long after the date of transplantation. These patients also had a better response to the COVID-19 vaccine (53). The conclusion that TTV is lower in patients who were transplanted longer ago, was also drawn by others in both KTR and LTR, confirming that TTV load predicted the response to the COVID-19 vaccine (54, 55). These cross sectional studies suggest that a plateau phase lasting for several years is debatable, but equally support the hypothesis that a high TTV load in the long term still reflects over-immunosuppression. A low TTV load nevertheless may over years become less predictive for the risk of rejections (47).

## Discussion and outlook

Two decades after the discovery of TTV as a first representative of the human virome, insights on how this virus can be used to assess the function of the immune system have evolved rapidly. Accumulating evidence points at the potential use for TTV in immunocompromised patients. SOTR may benefit most from a marker of immunosuppression, as in this population the degree of immunosuppression is amendable and can be adjusted to a desired level. As to what constitutes a desired level will have to be answered by clinical studies, such as the ones which are currently conducted. Both these two clinical studies use the commercially available TTV-quantitative PCR test in their protocols, and exclude participants with consistently negative TTV loads after transplantation, thereby overcoming some of the limitations detailed above. While the VIGILung trial focusses on TTV as a predictor of toxicity associated with high dosages of tacrolimus, the TTV GUIDE IT study aims to examine the immunosuppressive status of the SOTR during the first few months after transplantation, similar to most of the longitudinal studies which have been published to date. The currently available data indicates a significant correlation between rejection and low TTV loads during precisely this period (Table 3). In contrast, the relationship between high TTV loads and risk for infection is less clear from existing evidence (3). Although there are several reports showing a correlation, infections after transplantation are not always caused by over-immunosuppression alone and their incidence can sometimes be decreased by use of prophylactic medication, as is the case for CMV infections (3, 7, 8). In the long term, over-immunosuppression may well represent the highest risk to the majority of transplant recipients (7). Dose-dependent side effects of antirejection drugs, as well as infections have a huge impact on the quality of life of transplant recipients and continuing graft survival (7, 49, 56). It is conceivable that TTV load could also be used as a marker to adjust immunosuppression 5, 10, or 20 years after transplantation, but there is as yet not enough evidence supporting that hypothesis. Barring a small number of cross-sectional studies, there are no studies investigating the use of TTV load measurement for longer than three years after transplantation. Such studies are still needed to investigate if a particular TTV load signifies the same risks several years after transplantation, or whether the ideal TTV load is changes in time. Aside from research in the organ transplant population which seems to be gaining momentum, more research is in general needed to answer remaining questions regarding the prevalence of TTV, the meaning of a negative TTV load, genotypic variation and detection of these genotypes by different assays, as well as replication of TTV and the immune response to this virus. Nevertheless, the two clinical studies into the use of TTV in the follow-up of SOTR represent an exciting new way of thinking in which the virome is used as a tool to assess the immune system.

## Author contributions

EG contributed to collecting articles, appraising references, and correcting the first draft of the manuscript. LG contributed to writing paragraphs with molecular data and critically reading and correcting the manuscript. HN contributed to critically reading the manuscript and editing. CV contributed to drafting the first manuscript and appraising references. All authors contributed to the article and approved the submitted version.
